# Development and Validation of a New Near-Infrared Sensor to Measure Polyethylene Glycol (PEG) Concentration in Water

**DOI:** 10.3390/s17061354

**Published:** 2017-06-10

**Authors:** Olivier Buzzi, Shengyang Yuan, Benjamin Routley

**Affiliations:** 1 Priority Research Centre for Geotechnical and Materials Modelling, University of Newcastle, Callaghan NSW 2308, Australia; Shengyang.Yuan@uon.edu.au; 2 School of Electrical Engineering and Computer Science, University of Newcastle, Callaghan NSW 2308, Australia; ben.routley@newcastle.edu.au

**Keywords:** fibre optic sensor, distributed feedback laser, polyethylene glycol (PEG), near-infrared

## Abstract

A near-infrared absorption based laser sensor has been designed and validated for the real-time measurement of polyethylene glycol (PEG) concentration. The wavelength was selected after the determination of the absorption spectrum of deionised water and PEG solutions using a Varian Cary 6000i spectrophotometer, in order to limit the influence of PEG molecular mass on the absorption measurement. With this new sensor, the water is treated as the attenuating species and the addition of PEG in water reduces the absorbance of the medium. The concept was validated using three different PEG types (PEG 6000, 20,000, and 35,000) and it was found that the results follow Beer Lambert’s law. The influence of temperature was assessed by testing the PEG 20,000 at four different temperatures that could be encountered in a laboratory environment. The data show a slight temperature influence (increase of absorbance by 8% when the temperature rises from about 20° to about 29°). Following the validation phase conducted ex situ, a prototype of an immersible sensor was built and calibrated for in situ measurements.

## 1. Introduction

Polyethylene glycol (PEG) is a water-soluble polymer of chemical formula C_2n_H_4n+2_O_n+1_ that is widely used in medical, biomedical, environmental and engineering applications. In particular, it is often employed to create very high osmotic pressures in biochemistry, bio membranes engineering, or geotechnical engineering [1]. Polyethylene glycol has also been deployed as a medium for dissolved oxygen concentration measurement [2], humidity sensors, and chronic glucose monitoring in vivo [3].

Because PEG is non-ionic or inert [3], measuring its concentration by standard chemistry techniques (e.g., electrical conductivity) is not possible. Researchers and scientists resort to a number of advanced techniques such as spectrophotometry (with or without chemical treatment) [4,5] or colorimetric methods [6]. Cheng et al. [7] recently provided a review of the various techniques possible to measure PEG concentration. All of these methods require advanced equipment and are not suited for continuous monitoring of PEG concentrations. To date, the simplest method to infer PEG concentration relies on the measurement of the refractive index of the PEG solution, a method that was proposed by Lagerweff et al. [2]. Although the method is simple, accurate equipment to measure the refractive index can be quite costly.

Consequently, the present study aims at developing a simple alternative method to measure PEG concentration based on the measurement of absorbance via a tunable diode laser. The principle of optical absorption has been used in a range of fields including combustion diagnostics [8], engine exhaust monitoring [9], analysis of landfill gases [10], and explosives detection [11]. It is also a method used to detect chemical species diluted in a solute [12,13]. Inferring concentrations from absorbance measurements relies on Beer Lambert’s law, which states that the absorbance is directly proportional to the concentration of attenuating species present in water. This technique can provide a simultaneous, non-intrusive, and reliable method for ex situ and in situ measurements.

Following UV-Vis measurements of the absorption spectrum of deionised water and different PEG solutions, a wavelength was selected for the new sensor in order to reduce the influence of PEG molecular mass on the measurement of its concentration. The sensor was then validated against a range of PEG solutions. The design is cost effective; it requires a limited number of components (with simultaneous multipoint measurements capability) and simple processing techniques. The paper presents the validation of the sensor, the estimation of errors, and the temperature sensitivity. Note that the present sensor was primarily developed for applications in the field of unsaturated soil mechanics, where simple solutions of PEG in deioinised water are used to apply osmotic pressure [14]. It is out of the scope of this paper to validate the sensor for more complex chemical solutions where PEG may interact with other species.

## 2. Materials and Methods

### 2.1. Rationale and Novelty

**Figure 1:**
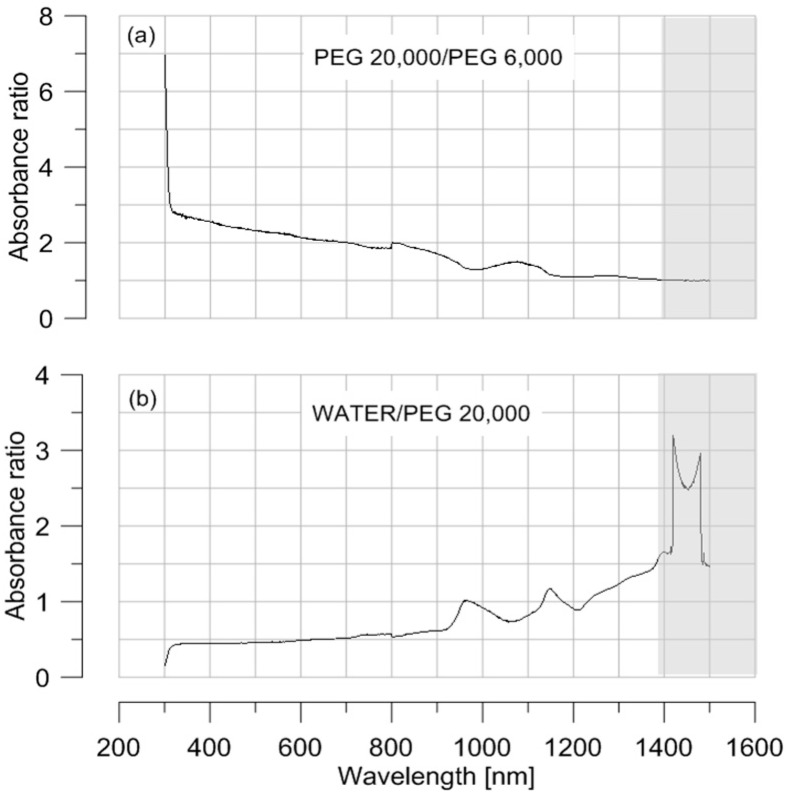
(**a**) Absorbance of the PEG 20,000 solution over absorbance of the PEG 6000 solution from 300 nm to 1500 nm. Solution concentration is 0.5 g PEG/g water; (**b**) Absorbance of deionised water over absorbance of PEG 20,000 (at 0.5 g PEG/g water) from 300 nm to 1500 nm.

The absorption spectrum of deionised water and two PEG solutions (PEG 6000 and PEG 20,000 both at 5 g PEG/g water) were obtained using a Varian Cary 6000i spectrophotometer (from Agilent Technologies), a high-performance InGaAs detector optimized for the shortwave (Near Infra Red) NIR, covering a wavelength range of 175 to 1800 nm. Results show that absorption of the PEG solutions is influenced by the PEG molecular mass up to infrared. From 1350–1400 nm (zone highlighted in light grey in Figure 1), the response of the PEG 6000 and PEG 20,000 solutions are very similar (ratio of 1, Figure 1a). Also of interest here is the fact that, for the infrared wavelengths, deionised water absorbs more than the PEG solutions (Figure 1b). In light of these absorption results, it has been decided that (1) near-infrared wavelength should be used in order to measure the PEG concentration without the influence of the PEG molecular mass; and (2) water concentration rather than PEG concentration should be detected since water absorbs more than the PEG. In other words, water is treated as the attenuating species. This approach goes against the mainstream applications of absorption measurement to detect species in solute, such as salinity [12] or proteins [13].

### 2.2. Experimental Setup

**Figure 2:**
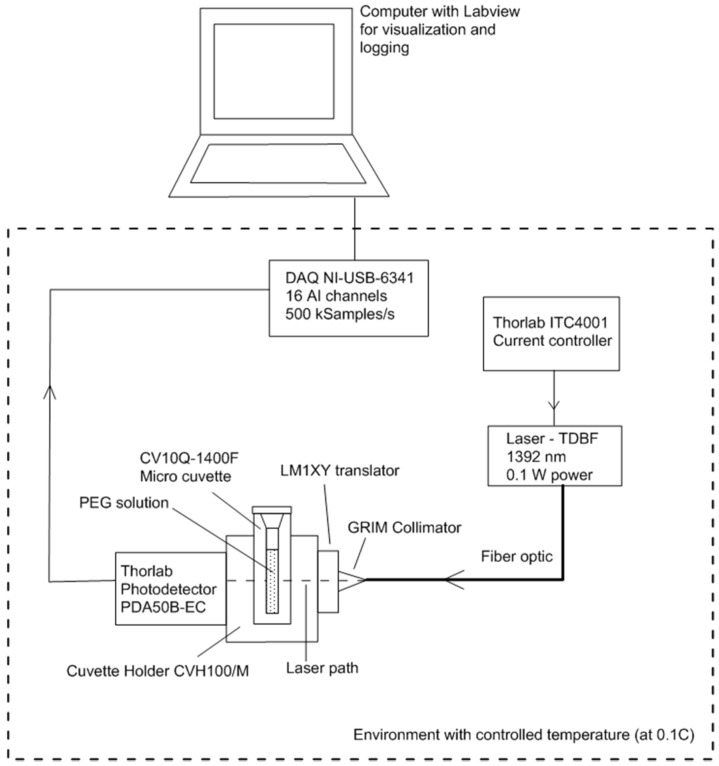
Sketch of the experimental setup used to validate the new sensor. The dashed line represents the boundary of the controlled temperature environment.

The experimental setup is described in Figure 2 and consists of three major parts: an emitting part, a sensing region and a receiving part. The emitting part includes a tunable distributed feedback (DBF) diode laser with 1392 nm central wavelength (from Frankfort laser company) mounted on an universal 14 pin butterfly LD Mount (Thorlabs model LM14S2) and a laser controller (Thorlabs model ITC4001), sending a constant current (of 42 mA) to the laser. The DFB laser is a fibre pigtailed laser with an internal isolator and course tuning range of 3 nm via Peltier temperature cooling element. The laser can be scanned over the entire wavelength range with a minimum resolution of 0.001 °C and 0.1 mA by temperature and current controller, respectively.

The sensing region consists of a cuvette holder (CVH100/M by Thorlabs) in which micro cuvettes (model CV10Q-1400F by Thorlabs) containing the PEG solutions can be inserted. With such an arrangement, the laser travels through 4 mm of PEG solutions and 8.5 mm of UV-fused quartz. The cuvettes were manipulated with gloves, cleaned after each experiment using deionised water, and dried with compressed air. As for the receiving part, it consists of a photodetector (460 kHz bandwidth and 800–1800 nm detection range, Thorlabs model PDA 50B-EC) mounted on the side of the cuvette holder. A collimator was used to align the laser travelling through an optic fibre and the photodetector.

Finally, the whole setup, apart from the computer, was placed in a laboratory fridge in order to maintain a constant temperature at ±0.1 °C (represented by the dashed line in Figure 2).

The signal coming from the photodetector was logged using a National Instrument Data Acquisition Unit at 500,000 samples/s. The voltage of the input (I_o_) and output (I) signals are used to compute the absorbance of the composite glass-PEG medium:

(1) $$A=\log_{10}\left(\frac{I_{o}}{I}\right)$$

In the following, all results will be expressed in terms of absorbance of the composite glass-PEG medium, which will be shown to vary with the PEG concentration.

### 2.3. PEG Solutions Tested

PEG comes in different molecular masses, depending on the length of the molecular chain (this is reflected by the *n* value in the chemical formula given in the Introduction). The choice of molecular mass largely depends on the intended application. In geotechnical engineering, values ranging from 6000 to 35,000 g/mol are common.

In order to assess whether the performance of the new sensor is affected by the PEG molecular mass, solutions of PEG 6000, PEG 20,000, and PEG 35,000 were prepared. For each PEG molecular mass, concentrations of 0.05, 0.1, 0.2, 0.3, 0.4, and 0.5 g PEG/g of water were prepared and tested. Higher concentrations were not tested as the viscosity becomes too high to be workable. Furthermore, at a given concentration, the viscosity of a PEG solution increases with its molecular mass. For this reason, the PEG 35,000 was only tested up to 0.4 g PEG/g H_2_O. The solutions were prepared by dissolving a determined mass of PEG in about 20 mL of deionised water. Masses were recorded using a high precision scale (to the 1/10,000th of a gram), hence minimizing the error on the concentration.

In the following, PEG 20,000 was used to assess the error and temperature dependence while all three PEG types were used to provide a correlation between absorbance and concentration. PEG 20,000 was also used to calibrate the immersible sensor to be presented in Section 4.

## 3. Results

### 3.1. Absorbance Data and Correlation with PEG Concentrations

**Figure 3:**
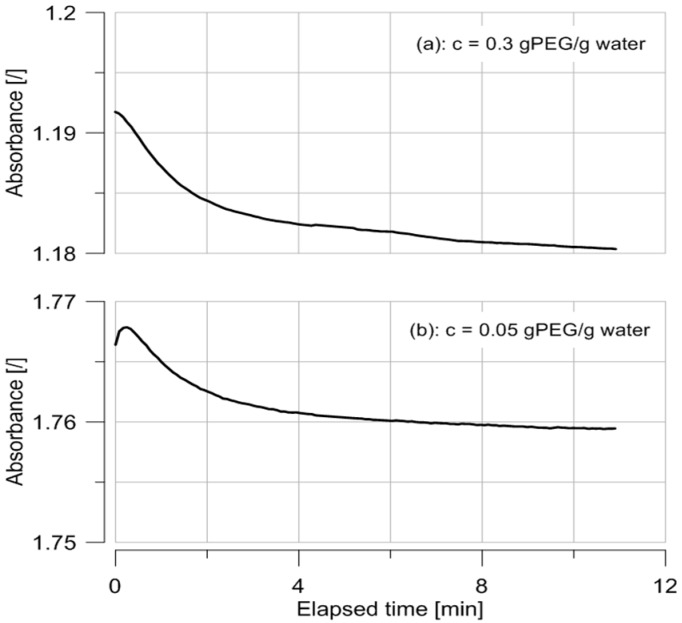
Evolution of absorbance with time of solutions of PEG 20,000 at 0.05 (**a**), and 0.3 g PEG/g water (**b**).

Figure 3 shows the evolution of absorbance in time for two concentrations of PEG 20,000. The equilibrium is reached after a few minutes and it can be noted that the absorbance decreases as the PEG concentration increases. This equilibration phase is attributed to thermal equilibration of the micro cuvettes that were kept outside the fridge. The results are consistent with the fact that water is the attenuating species. In other words, for a constant volume, the more PEG, the less water and the lower the absorbance.

**Figure 4:**
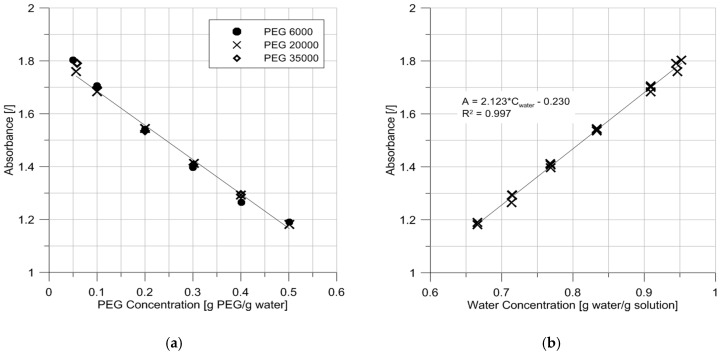
(**a**) Evolution of absorbance with PEG concentration for three different PEG molecular masses; (**b**) Evolution of absorbance with water concentration (no distinction of PEG types). Tests were performed at 20.5 °C.

Figure 4a presents the results obtained for the three PEG types and for all concentrations. It clearly shows a linear relationship between absorbance and PEG concentration as well as no effect of the PEG molecular mass, which was expected by opting for a near-infrared wavelength (see Figure 1a).

The magnitude of absorption is related to the concentration of the attenuating species, which is captured by Beer Lambert’s law:

A = a × b × c(2)

where A is the absorbance (no unit), a is the attenuation coefficient (L/mol/cm), b is the path length (cm) and c is the concentration of the attenuating species (mol/L). The proportionality between absorbance and concentration appears when plotting the absorbance results against the concentration of water (Figure 4b). One can notice a small intercept in the equation of the best fit, which is attributed to the variability in the cuvettes’ absorption. Figure 4 fully validates the concept of the new sensor, which can be used to measure PEG concentration in water.

Since the purpose of the sensor is to infer the PEG concentration, in the following, results will be presented as a function of PEG concentration rather than as a function of the concentration of the attenuating species (i.e., water).

### 3.2. Errors and Uncertainties

**Figure 5:**
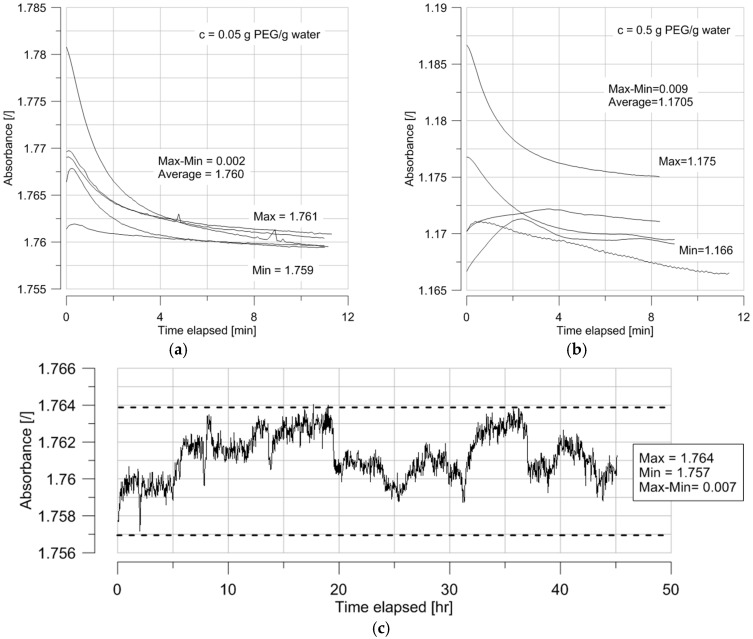
(**a**) Repeated absorbance measurements for PEG 20,000 at 0.05 g PEG/g water; (**b**) Repeated absorbance measurements for PEG 20,000 at 0.5 g PEG/g water; (**c**) Continuous measurement of PEG 20,000 at 0.05 g PEG/g water over a 45-h period. All tests were performed at 20.5 °C.

This section details the estimation of uncertainties and errors on the absorbance measurement and on the estimation of PEG concentration using the ex situ setup. The resolution of the measurement is 1/1000th of absorbance. However, the repeatability and possible drift have to be considered for a proper quantification of the error. Indeed, the intended application, namely unsaturated soil testing, implies continuous measurement over several days. Figure 5a,b show the variability of the response for the lowest and highest PEG concentrations when measurements are repeated on the same solution sample. The highest difference between measurements occurs for the highest PEG concentration (Figure 5b) and is about 0.009. The reasons for this variability are not fully identified, but could include slightly different positions of the micro-cuvettes or the presence of air nuclei in the solution.

Logging the absorbance of PEG 20,000 at 0.05 g PEG/g H_2_O over 45 h showed no drift but small fluctuations in the absorbance data, with an amplitude of about 0.007, which are attributed to the accuracy of temperature control of the laboratory fridge.

The uncertainty intervals pertaining to resolution, repeatability, and temperature fluctuation are independent (i.e., come from different sources) but can be combined in order to obtain an overall equivalent resolution $\Delta A_{equivalent}$:

(3) $$\begin{array}{l} \Delta A_{equivalent}=\sqrt{\Delta A_{resolution}^{2}+\Delta A_{repeat}^{2}+\Delta A_{fluctations}^{2}}=\sqrt{{\left(\pm 0.001\right)}^{2}+{\left(\pm 0.0045\right)}^{2}+{\left(\pm 0.0035\right)}^{2}}\\ \Rightarrow \Delta A_{equivalent}=\pm 0.0057 \end{array}$$

**Figure 6:**
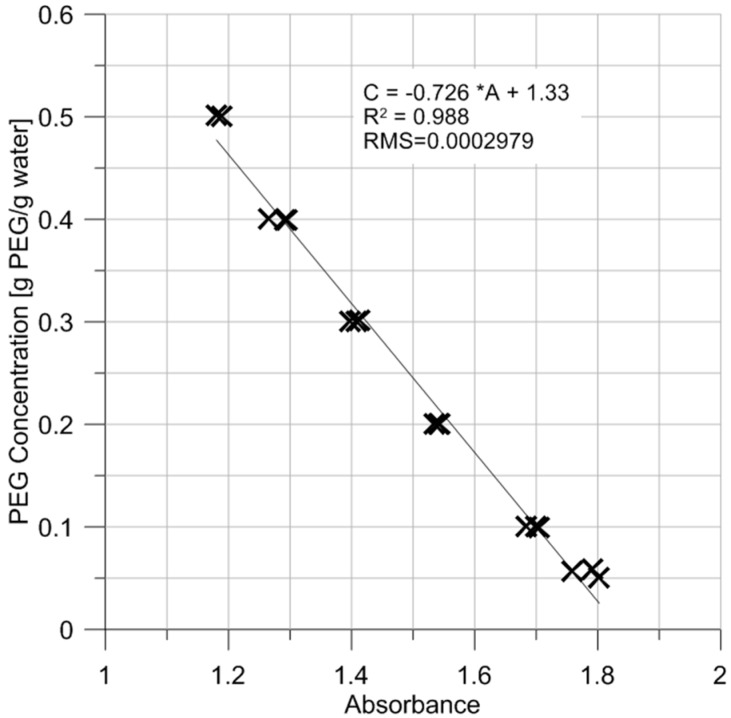
PEG concentration as a function of absorbance for all tests performed. All measurements were conducted at 20.5 °C.

Another source of errors to be herein considered pertains to the goodness of fit of the absorbance—concentration data. The linear curve fitting presented in Figure 6 for all absorbance data returns an R^2^ of 0.988 and a residual mean square of 2.979 × 10^−4^.

The standard error being the square root of the residual mean square, it yields an absolute error of 0.017 g PEG/g water, which represents about 34% and 3.4% of relative error for PEG concentrations of 0.05 and 0.5 g PEG/g water, respectively. This error is relatively high and is due to data scattering. It has to be borne in mind that the absorbance values pertain to the PEG solution and the cuvettes. It was found afterwards that the empty cuvettes do not all have the same absorbance; the values were found to range between 0.028 and 0.078, which could contribute to the scattering.

### 3.3. Sensitivity to Temperature

The results presented so far were all obtained at a temperature of 20.5 °C. In this section, the influence of temperature was ascertained by repeating the measurements under temperatures of 23.5, 26.2, and 29.5 °C. Such temperatures have been reached in the Civil Laboratory of the University of Newcastle in summer. For the sake of clarity, the data are presented without error bars; however, this does not compromise the discussion and conclusions because the magnitude of changes observed is larger than the error.

**Figure 7:**
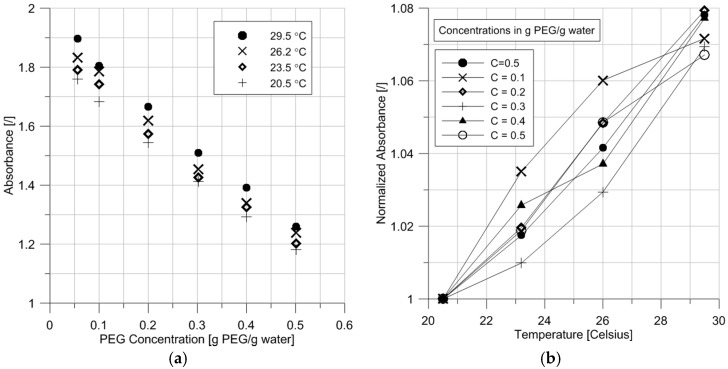
(**a**) Evolution of absorbance of PEG 20,000 solutions as a function of PEG concentration for different temperatures; (**b**) Evolution of the normalized absorbance, defined as the absorbance at temperature T over the absorbance at 20.5 °C, with temperature.

A clear and consistent trend appears in Figure 7a with the absorbance of the PEG solutions increasing with temperature. The linear relationship between absorbance and concentration is maintained but it is progressively shifted towards higher values of absorbance. Figure 7b shows the same data, expressed in terms of normalized absorbance, defined as the absorbance at temperature T over the absorbance at 20.5 °C. Augmenting the temperature consistently translates into an increase in absorption, which is related to the temperature sensitivity of the water absorption spectrum. Collins [15] showed that an increase of temperature could increase or decrease the magnitude of absorption, depending on the wavelength used.

The results show that the temperature dependence should be accounted for when calibrating the sensor and that, consistent with the general usage of the osmotic technique, an adequate control of temperature is required [16,17,18].

Davies and Shields [5] used spectrophotometry to estimate the concentration of PEG 4000 in tyrode solution. They obtained a linear relationship between absorbance and PEG concentration with a rise of absorbance as the PEG concentration increases. This finding contradicts the trend observed here, but this is likely due to different wavelengths. Indeed, PEG does not absorb at wavelength 1392 nm but it does absorb at 3472 nm. This means that, depending on the wavelength, it is either a non-attenuating species or an attenuating species. Davies and Shields did not report accuracy readings, but Selisko et al. [19] found detectable concentrations of 1.6 μg/mL and 1.7 μg/mL for PEG 4100 and PEG 8650, respectively. Guermant et al. [20] found a detectable concentration of 0.1 μmol/L for mPEG 5000. Such detectable concentrations are much lower than the values found, which can possibly be explained by the fact that the studies cited above relied on high accuracy spectrometers. In contrast, several components were here assembled to measure absorbance in order to reach the objective of developing a sensor for in situ measurement (see Section 4). Finally, Guermant et al. [20] stated that their calibration curve is a function of the mPEG molecular weight, but Figure 4a shows that using a wavelength of 1392 nm renders the calibration independent of the PEG molecular weight.

## 4. Construction and Calibration of a Prototype in Situ Sensor

**Figure 8:**
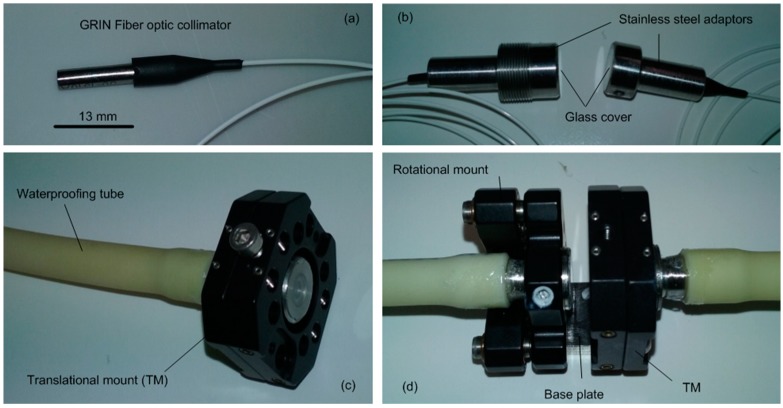
Components of the immersible sensor for the in situ measurement of PEG concentrations. (**a**) Optic fibre connected to a GRIN collimator; (**b**) Emitting and receiving GRIN collimators placed in stainless steel adaptors with glass cover for water tightness.; (**c**) Stainless steel adaptor connected to a translational mount used to adjust the relative positions of the GRIN collimators and ensure that the laser enters the receiving GRIN collimator. A waterproof tube is used for water tightness; (**d**) Both GRIN collimators, installed in the translational and rotational mounts. Both mounts are fixed to a base plate maintaining the position of the mounts and the gap between the two GRIN coolimators.

**Figure 9:**
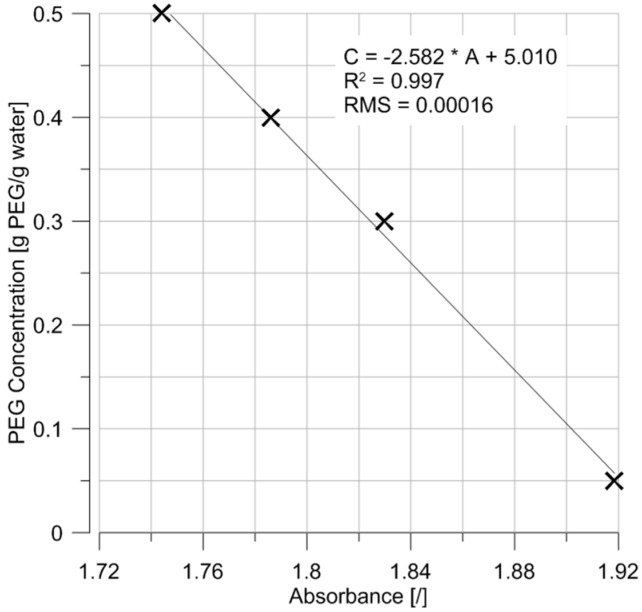
Preliminary calibration curve of the immersible sensor for PEG concentration measurement.

Following the proof of concept presented in the previous sections, an immersible sensor was built to allow the in situ measurement of PEG concentrations. The various components of the sensor are presented in Figure 8, and a preliminary calibration of the sensor is presented in Figure 9.

The laser beam shines through a first GRIN fibre optic collimator (Figure 8a) and is collected via a second collimator. These collimators are placed in stainless steel adaptors (Figure 8b) that are themselves positioned in aligning mounts (Figure 8c,d). Using both a translational and a rotational mount is required to align both collimators and ensure that the laser beam can be detected.

Optic fibres can be damaged if immersed in water, thus the watertightness of the sensor is critical. This was achieved via a combination of plastic tubes, thread sealant, and glass covers glued at the end of the stainless steel adaptors. The gap between the collimators is about 6 mm (maintained constant by fixing both mounts on a stainless steel baseplate), including about 5 mm between the glass covers. As per Section 2.2, one end of the sensor was connected to the current controller for current supply (constant current of 42 mA) while the other end was connected to the photodetector. The sensor was immersed in a number of PEG 20,000 solutions of controlled concentration (prepared as per Section 2.3) under a constant temperature of 20.5 °C.

The correlation between PEG concentration and measured absorbance, in other words the calibration curve of the sensor, is showed in Figure 9. Due to technical issues with the controller (still to be resolved), only these four data points were obtained. Nonetheless, the points confirm the linear correlation between absorbance and concentration and the preliminary calibration curve of the immersible sensor can be established as:

C = −2.582 × A + 5.010(4)

Note that, for a given concentration and at a temperature of 20.5 °C, the absorbance values in Figure 9 are higher than those presented in Figure 6, which is explained by a longer laser path through the PEG (“b” term in Beer Lambert’ law, see Equation (2)).

## 5. Conclusions

This paper presents a new device to measure the concentration of PEG in water in the context of unsaturated soil testing. The proof of concept was validated after a first series of tests demonstrating that it is possible to use the water as the attenuating species, and that a linear correlation can be obtained between the concentration of PEG and the absorbance of the signal. Interestingly, the correlation does not depend on the PEG molecular weight, meaning that the calibration factor of the sensor does not need adjustment for PEG molecular weight. The errors and uncertainties were estimated, and it was found that the resolution of the sensor is ±0.0057 [no unit]. Although the resolution of absorbance measurement is good, the residual mean square of the calibration curve is 2.979 × 10^−4^, which translates into a relative error of about 34% at low concentration. Such a value of error is quite high and further research is required to reduce the error. Finally, the effect of temperature was ascertained. It was found that the absorbance increases by about 8% as the temperature increases from 20.5 °C to 29.5 °C. The paper concludes with the presentation of a prototype sensor for in situ measurement and its preliminary calibration curve. Such technology opens the door for automated monitoring of PEG solutions in unsaturated soil testing, which would avoid the tedious process of manual measurement. In addition, because the wavelength selected is fully compatible with optic fibres, it is possible to use a network of sensors deployed in parallel from only one laser source. This new sensor could easily be used in other fields such as biomedical sciences, the food industry, and the pharmaceutical industry.
